# MTA1-Dependent Anticancer Activity of Gnetin C in Prostate Cancer

**DOI:** 10.3390/nu11092096

**Published:** 2019-09-04

**Authors:** Avinash Kumar, Kshiti Dholakia, Gabriela Sikorska, Luis A. Martinez, Anait S. Levenson

**Affiliations:** 1 Arnold & Marie Schwartz College of Pharmacy and Health Sciences, Long Island University, Brooklyn, NY 11201, USA (K.D.) (G.S.); 2 Stony Brook School of Medicine, Stony Brook, NY 11794, USA; 3 School of Veterinary Medicine, Long Island University, Brookville, NY 11548, USA

**Keywords:** Gnetin C, resveratrol, pterostilbene, MTA1, ETS2, prostate cancer

## Abstract

The overexpression of metastasis-associated protein 1 (MTA1) in prostate cancer (PCa) contributes to tumor aggressiveness and metastasis. We have reported the inhibition of MTA1 by resveratrol and its potent analog pterostilbene in vitro and in vivo. We have demonstrated that pterostilbene treatment blocks the progression of prostatic intraepithelial neoplasia and adenocarcinoma in mouse models by inhibiting MTA1 expression and signaling. In the current study, we investigated the MTA1 targeted anticancer effects of Gnetin C, a resveratrol dimer, in comparison with resveratrol and pterostilbene. Using DU145 and PC3M PCa cells, we found that Gnetin C downregulates MTA1 more potently than resveratrol and pterostilbene. Further, Gnetin C demonstrated significant MTA1-mediated inhibitory effect on cell viability, colony formation, and migration, while showing a more potent induction of cell death than resveratrol or pterostilbene. In addition, we identified Gnetin C-induced substantial ETS2 (erythroblastosis E26 transformation-specific 2) downregulation, which is not only MTA1-dependent, but is also independent of MTA1 as a possible mechanism for the superior anticancer efficacy of Gnetin C in PCa. Together, these findings underscore the importance of novel potent resveratrol dimer, Gnetin C, as a clinically promising agent for the future development of chemopreventive and possibly combinatorial therapeutic approaches in PCa.

## 1. Introduction

Prostate cancer (PCa) is a major public health issue in the U.S. It is reported that about 175,000 men will be diagnosed with PCa and around 32,000 will die from the disease in 2019 [1]. Along with the biggest challenges for urologists and oncologists, which include differentiation between indolent and aggressive PCa and finding treatment for metastatic disease, there is a major problem of the management of a large number of patients that are usually recommended for active surveillance or “watchful waiting”. These patients are with high prostate-specific antigen (PSA) levels and negative biopsies or with positive biopsies of low Gleason scores. Current management of these patients involves monitoring PSA levels, repeat biopsies, and no active treatment. Unfortunately, about 40% of these patients develop PCa and/or progress to advanced stages, posing a significant demand for urgent and effective chemopreventive measures.

Most PCa cases are not hereditary: 90–95% of cases are acquired over an individual’s lifetime because of an influence of environmental factors, including risk factors such as age, obesity, and diet. While other risk factors such as age or ethnicity are immutable, diet can be easily changed and controlled. Epidemiological and observational data endorse diet as a risk factor for PCa [2]. Attempts were made to prevent PCa by dietary bioactive compounds, but they mostly failed [3,4,5,6]. In general, dietary bioactive molecules are pleotropic, i.e., they affect many signaling pathways. Therefore, it is important to “personalize” chemopreventive strategies by defining the particular subset of patients that is likely to respond to certain dietary agents via specific signaling pathways.

The overexpression of chromatin modifier protein, MTA1 (metastasis-associated protein 1), in PCa contributes to tumor aggressiveness and metastasis [7,8,9]. We have reported previously on the important role of MTA1 and MTA1-associated signaling at all stages of PCa progression [10], including its critical role in bone metastasis [8,9,11]. Our earlier MTA1 chromatin immunoprecipitation sequencing (ChIP-Seq) analysis identified downstream targets that are transcriptionally regulated by MTA1 and suggested a link between MTA1 and ETS2 (erythroblastosis E26 transformation-specific 2) transcription factor [12]. The effects of ETS2 in cancer are context-dependent, and both oncogenic and tumor suppressive functions have been described [13,14,15,16,17,18,19,20,21,22,23,24,25,26,27]. We observed a positive correlation between MTA1 and ETS2 using loss of function studies and preclinical models of PCa [12].

In our previous studies, we have extensively reported on the anticancer properties of resveratrol (*trans*-3,5,4′-trihydroxystilbene, Res) and its natural analogs, specifically pterostilbene (*trans*-3,5-dimethoxy-4-hydroxystilbene, Pter), through their potent targeting of MTA1-associated tumor promoting signaling in PCa in vitro and in vivo [12,28,29,30]. We have also demonstrated that trimethoxy-resveratrol and piceatannol when administered orally suppress tumor formation and growth in PCa xenografts [31]. In addition, pterostilbene treatment blocks the progression of prostatic intraepithelial neoplasia (PIN) and adenocarcinoma in xenografts and transgenic mouse models by inhibiting MTA1 expression and signaling [12,30], suggesting superior pharmacological potency for resveratrol’s natural analogs with improved pharmacokinetics. When pterostilbene was tested in combination with histone deacetylase (HDAC) inhibitor SAHA (suberoylanilide hydroxamic acid, vorinostat), tumor tissues from prostate-specific Pten-null mice showed a reduction of MTA1-associated proangiogenic factors HIF-1α, VEGF, and IL-1β leading to decreased angiogenesis and more potent antitumor effects [32].

In the current study, we evaluated the superior MTA1-dependent anticancer effects of Gnetin C, which is a resveratrol dimer that is found abundantly in melinjo plant (*Gnetum gnemon*) that is widely cultivated and used in Indonesian culinary, compared to resveratrol and pterostilbene. Overall, our data define the anticancer mechanisms of Gnetin C through suppression of the MTA1/ETS2 axis and the effect on the MTA1-mediated clonogenic, migrative, and proliferative capacity of PCa cells. These findings suggest that Gnetin C has potent anticancer activity in PCa and supports the chemopreventive and therapeutic potential of this relatively less studied dietary stilbene.

## 2. Materials and Methods

### 2.1. Reagents

Resveratrol and pterostilbene were purchased from Sigma-Aldrich (St. Louis, MO, USA). Gnetin C was a generous gift from Hosoda SHC Co., Ltd. (Fukui, Japan). All compounds were dissolved in dimethyl sulfoxide (DMSO) such that final concentration was 0.1% DMSO.

### 2.2. Cell Culture

Prostate cancer cell, PC3M, and DU145, were cultured in RPMI-1640 media containing 10% fetal bovine serum (FBS) as described recently [11,33]. A549 lung adenocarcinoma cells were cultured in RPMI-1640 media containing 10% FBS as described previously [34] and were used only for transfection experiments. MTA1 knockdown PC3M and DU145 cells were generated as described previously using three different shRNAs and the clone with most efficient knockdown was chosen for consequent and current studies [11]. Cells were maintained in an incubator at 37 °C with 5% CO2. Cells were authenticated at the Research Technology Support Facility, Michigan State University by using short tandem repeat profiling.

### 2.3. Plasmids and Transfections

HA-ETS2 and Myc-ETS2 constructs were used by us previously [34]. The Myc-MTA1 construct was a kind gift from Dr. Guri Tzivion, Windsor University School of Medicine, Brighton’s Estate, Cayon, St. Kitts. For transfections, A549 or MTA1 knockdown DU145 and PC3M cells were plated to reach 80–90% confluence and were transfected with Myc-MTA1, Myc-ETS2, and HA-ETS2 in OptiMEM media (ThermoFisher Scientific, Waltham, MA, USA) using Fugene HD (Promega, Madison, WI, USA). After 48 h, cells were harvested and immunoprecipitation (IP), Western blot, qRT-PCR, or wound-healing assay procedures were followed, as described in the following sections.

### 2.4. Western Blotting

Western blotting was performed as described earlier [11,33]. After cells were treated with various concentrations of Res, Pter or Gnetin C for 24 h, total protein was isolated and estimated using Bio-Rad protein assay reagent. Protein samples were separated in 10% polyacrylamide gels, transferred onto polyvinylidene difluoride (PVDF) membranes, blocked with 5% milk–PBS–0.1%Tween, and then probed with primary antibodies listed in Supplementary Table S1. β-actin was used as a loading control. Signals were detected using enhanced chemiluminescence. Bands intensity was measured using Image J (NIH, Bethesda, MD, USA).

### 2.5. qRT-PCR

RNA was isolated using an RNeasy Mini Kit (Qiagen, Hilden, Germany) and reverse transcribed with SuperScript II Reverse Transcriptase. qRT-PCR was performed as described earlier on the Lightcycler 480 II Real-Time PCR instrument (Roche Diagnostics, Basel, Switzerland) [11]. mRNA expression was determined relative to β-actin using the primers listed in Supplementary Table S2.

### 2.6. MTT Assay

MTT cell proliferation assay (Sigma-Aldrich) was performed to assess the cell viability of parental and MTA1 knockdown DU145 and PC3M cells after treatment with vehicle, Res, Pter, or Gnetin C, as described earlier [32,33,35]. The cells were seeded into 96-well plates, and after 72 h of treatment, the absorbance was measured using a BioTek Synergy-4 plate reader (BioTek, Winooski, VT, USA). The viability of vehicle-treated control cells was assigned as 100%, and the percentage of cell viability of the treated cells was calculated on that basis. IC_50_ was calculated using GraphPad Prizm v7 (GraphPad Software, La Jolla, CA, USA).

### 2.7. Flow Cytometry

Cytotoxicity induced in parental and MTA1 knockdown DU145 and PC3M cells by treatment with Res, Pter, or Gnetin C was determined by flow cytometry. About 2 × 10^5^ cells were seeded in 60-mm culture dishes and treated with vehicle, Res, Pter, or Gnetin C. After 24 h of treatment, the cells were washed with cold PBS, fixed in chilled 95% ethanol, and incubated overnight at −20 °C. The next day, the cells were again washed with PBS and then stained with propidium iodide/RNase A staining solution at 37 °C for 30 min. The stained cells were passed through a cell strainer and run on the CytoFLEX flow cytometer (Beckman Coulter, Indianapolis, IN, USA). Acquisition and analysis were performed using the CytExpert 1.2 software.

### 2.8. Colony Formation Assay

Approximately 2 × 10^3^ parental non-silenced (NS) and MTA1 knockdown DU145 and PC3M cells were seeded in 35-mm culture dishes for the 2-week observation time, as described recently [11,33]. Cells were treated with vehicle, Res, Pter, or Gnetin C every other day. Upon the formation of colonies, cells were fixed and stained with 0.01% crystal violet solution. Images of colonies were captured using an Amersham Imager 600 and analyzed using ImageQuant TL software (GE Healthcare Bio-Sciences, Pittsburg, PA, USA). 

### 2.9. Wound Healing Assay

Treatment induced motility changes in parental (NS) and MTA1 knockdown DU145 and PC3M cells were determined by the wound healing assay as described earlier [11,33,36]. A total of 90% confluent cells were starved in media containing 0.1% serum overnight, after which wounds were scratched through the cells. Images of wounds were captured using the EVOS XL Core microscope (ThermoFisher Scientific, Waltham, MA, USA) and analyzed using the ImageJ (NIH, Bethesda, MD, USA). The percentage of wound area was quantitated, assuming 100% for vehicle-treated cells at 0 h.

### 2.10. Immunoprecipitation

A549 cells were transfected with either Myc-ETS2 or Myc-MTA1 alone or together (co-transfection) as described in the “Plasmids and transfections” section. Protein estimation was performed after harvesting the cells in IP lysis buffer. One mg protein was incubated with 2 μg of ETS2 antibody and rocked overnight at 4°C, followed by incubation with Protein G beads for 2 h at 4 °C. Beads were washed several times with lysis buffer, boiled in sample buffer, and analyzed using SDS-PAGE followed by immunoblotting as described in the “Western blotting” section using Myc primary antibody.

### 2.11. Immunofluorescence

DU145 cells that were 60–70% confluent were fixed using 2% chilled paraformaldehyde. Then, the cells were washed with cold PBS and permeabilized in 0.5% TritonX-100/PBS for 15 min. After washing and blocking, cells were incubated first with MTA1 antibody (A-11) followed by secondary antibody Alexa 488 in staining wash buffer (5% FBS/0.5% TritonX-100/PBS). This was followed by incubation with ETS2 antibody (E-5) and secondary antibody Alexa 633. Secondary antibodies (1:200) were purchased from ThermoFisher Scientific Waltham, MA, USA. Finally, cells were mounted in Vectashield with DAPI (4ˊ,6-diamidino-2-phenylindole) (Vector Laboratories, Burlingame, CA, USA). Unstained cells and cells incubated with secondary antibody alone served as negative controls. Images were collected using the Leica Brightfield Fluorescent microscope (Leica Microsystems, Buffalo Grove, IL, USA). Images were collected using a 63X objective.

### 2.12. Statistical Analysis

All data for each treatment group were summarized as the mean ± SEM. The differences between groups were determined by one-way or two-way ANOVA or the two-tailed two-sample t-test based on the experiment design. A p value of ≤0.05 was considered significant. All the experiments were performed at least three times. Statistical plots were generated using GraphPad Prism v7 (GraphPad Software, La Jolla, CA, USA).

## 3. Results

### 3.1. Gnetin C Inhibits MTA1 in PCa Cells More Potently than Resveratrol and Pterostilbene

**Figure 1:**
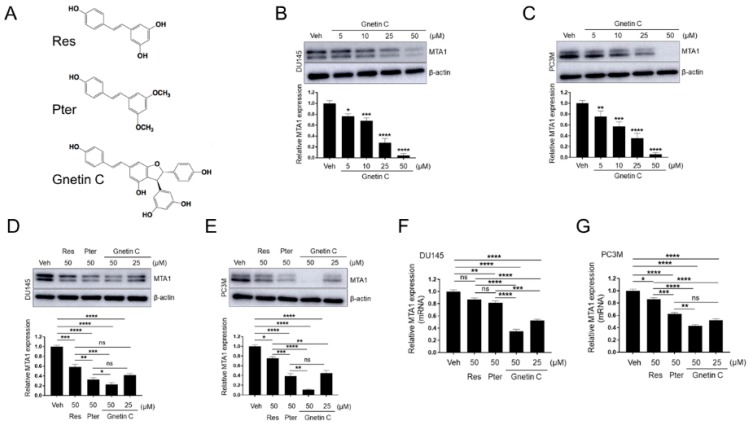
Gnetin C inhibits metastasis-associated protein 1 (MTA1) protein and mRNA expression in prostate cancer (PCa) cells more potently than resveratrol and pterostilbene. (**A**) Chemical structures of resveratrol, pterostilbene (Pter), and dimer-resveratrol (Gnetin C). (**B**,**C**) Dose-dependent inhibition of MTA1 protein expression by Gnetin C in DU145 and PC3M cells, respectively. *Bottom*, quantitation of MTA1 levels under Gnetin C treatment. (**D**,**E**) Comparison of MTA1 inhibition by Res (50 μM), Pter (50 μM), and Gnetin C (25 μM, 50 μM) in DU145 and PC3M cells, respectively. *Bottom*, quantitation of MTA1 levels under Res, Pter, and Gnetin C treatment. (**F**,**G**) MTA1 mRNA levels were analyzed by qRT-PCR after treatment with Res (50 μM), Pter (50 μM), and Gnetin C (25 μM, 50 μM) in DU145 and PC3M cells, respectively. Quantifications represent the mean ± SEM of three independent experiments. * *p* < 0.05; ** *p* < 0.01; *** *p* < 0.001; **** *p* < 0.0001 (one-way ANOVA).

In our previous studies, we have reported that Res but more so Pter inhibited MTA1 expression and showed anticancer and antimetastatic effects against PCa in vitro and in vivo [12,28,29,30,32,37]. To extend our observation on the stilbene-mediated downregulation of MTA1, we performed comparative analysis among Res, Pter, and Gnetin C, which is a natural resveratrol dimer (Figure 1A). We found that Gnetin C inhibited the expression of MTA1 in DU145 and PC3M cells in a dose-dependent manner (Figure 1B,C). When compared to Res and Pter applied at the same 50-μM concentration, Gnetin C demonstrated a significantly greater potent inhibition of MTA1 at both protein (Figure 1D,E) and mRNA (Figure 1F,G) levels in DU145 as well as PC3M cells. Notably, Gnetin C demonstrated significant inhibition of MTA1 at a twofold lower, 25 μM dose, which was comparable with effects of higher, 50 μM doses of Res and Pter (Figure 1D–G).

### 3.2. Gnetin C Induces MTA1-Dependent Cytotoxicity in PCa Cells more Potently than Resveratrol and Pterostilbene

**Figure 2:**
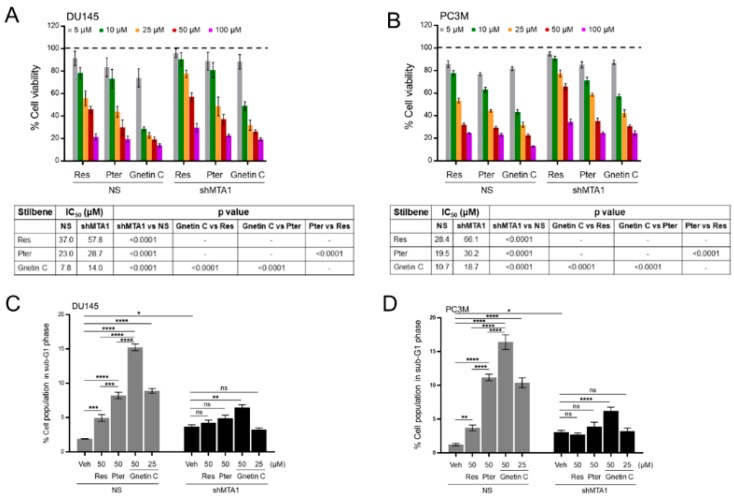
Gnetin C induces MTA1-dependent cytotoxicity in PCa cells more potently than resveratrol and pterostilbene. (**A**,**B**) Cell viability analysis of DU145 and PC3M cells treated with various concentrations (5–100 μM) of Res, Pter, and Gnetin C. *Bottom*, IC_50_ quantifications from the cell viability analysis. (**C**,**D**) Cell cycle analysis of DU145 and PC3M cells showing percentage of cell death after treatment with Res (50 μM), Pter (50 μM), and Gnetin C (25, 50 μM). Data represent the mean ± SEM of three independent experiments. * *p* < 0.05; ** *p* < 0.01; *** *p* < 0.001; **** *p* < 0.0001 (one-way ANOVA).

Due to the previously reported association between the expression levels of MTA1 and stilbene-induced anticancer activity in PCa [28,30], we measured the overall impact of MTA1 silencing in PCa cells on the sensitivity of cells to stilbene treatments. We performed cell viability assay in DU145 and PC3M cells expressing MTA1 (NS) and silenced for MTA1 (shMTA1) treated with Res, Pter, and Gnetin C. As shown in Figure 2A,B, all three compounds could induce cytotoxicity relative to the vehicle-treated PCa cells. The treatment of DU145 and PC3M cells expressing MTA1 (NS) with Res, Pter, and Gnetin C resulted in cytotoxic effects with Gnetin C exhibiting three to fivefold lower IC_50_ values compared to Res and Pter, demonstrating significantly more potent efficacy for Gnetin C in both cell lines. Notably, the treatment of shMTA1 cells resulted in an overall lower cytotoxic effect in both cell lines, as revealed by a corresponding significant increase of IC_50_ values compared to NS cells, suggesting the MTA1-mediated cytotoxic effect of all compounds (Figure 2A,B).

To determine the contribution of cell death to the MTA1-mediated cytotoxicity induced by stilbenes, we performed cell cycle analysis in NS and shMTA1 DU145 and PC3M cells using flow cytometry. All three compounds caused cell death in cells expressing MTA1 (NS), with Gnetin C being the most potent inducer of cell death (Figure 2C,D; Supplementary Figure S1). Markedly, half a dose of Gnetin C (25 μM) had more potent efficacy than Res at 50 μM and comparable efficacy with Pter at 50 μM in both cell lines. Moreover, MTA1 loss compromised the ability of Res and Pter but not Gnetin C to induce cell death, suggesting that the mechanism responsible for Res/Pter- induced cell death is MTA1-dependent, whereas Gnetin C induced cell death, at least in part, through mechanisms independent of MTA1 (Figure 2C,D; Supplementary Figure S1).

### 3.3. Gnetin C Inhibits MTA1-Dependent Metastatic Potential of PCa Cells More Potently than Resveratrol and Pterostilbene

**Figure 3:**
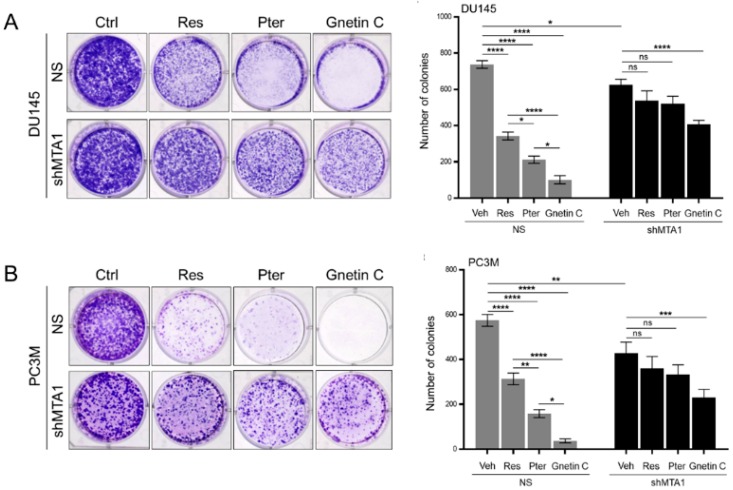
Gnetin C reduces MTA1-mediated clonogenic survival in PCa cells more potently than resveratrol and pterostilbene. (**A**,**B**) Representative images showing the colony formation ability of DU145 and PC3M MTA1 expressing (NS) and MTA1 knockdown (shMTA1) cells after treatment with 5 μM of Res, Pter, and Gnetin C. *Right*, Quantifications of the number of colonies using ImageQuant software are shown for each cell line. Data represent the mean ± SEM of three independent experiments with duplicate wells. * *p* < 0.05; ** *p* < 0.01; *** *p* < 0.001; **** *p* < 0.0001 (one-way ANOVA).

We next examined the MTA1-mediated effects of stilbenes on clonogenic survival. Analysis of the colony formation activity of cells cultured in the presence or absence of stilbene compounds revealed that stilbenes could induce a reduction of clonogenic survival compared to vehicle-treated DU145 and PC3M cells, with Gnetin C exhibiting significantly more potent effects compared to Res and Pter (Figure 3A,B). Notably, when MTA1 knockdown cells were treated with Res and Pter, the inhibitory effects were drastically reduced, suggesting their MTA1 dependency. However, Gnetin C demonstrated a significant reduction of clonogenic survival also in shMTA1 cells, signifying its partial MTA1 independent mechanism of action (Figure 3A,B).

**Figure 4:**
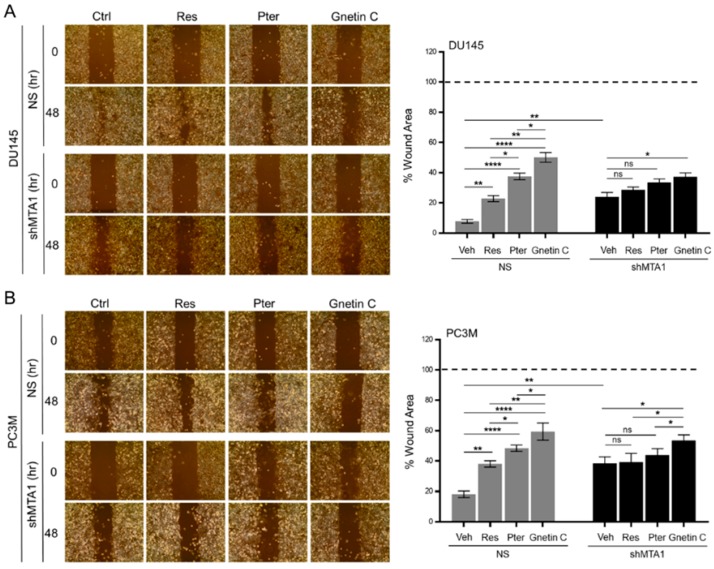
Gnetin C reduces the MTA1-mediated motility of PCa cells more potently than resveratrol and pterostilbene. (**A**,**B**) Representative images showing the migration ability of DU145 and PC3M MTA1 expressing (NS) and MTA1 knockdown (shMTA1) cells after treatment with 1 μM of Res, Pter, and Gnetin C for 48 h. *Right*, Quantifications of wound widths as a percentage of wound area using ImageJ software is shown for each cell line. Data represent the mean ± SEM of six separate wounds and three independent experiments. * *p* < 0.05; ** *p* < 0.01; *** *p* < 0.001; **** *p* < 0.0001 (one-way ANOVA).

We next sought to measure the MTA1-mediated effects of stilbene compounds on the migrative capacity of PCa cells. Analysis of wound healing assay of cells cultured in the presence or absence of stilbene compounds revealed that all three compounds could induce a reduction of the wound healing compared to vehicle-treated cells (Figure 4A,B). However, Gnetin C exhibited the most profound effects in both DU145 and PC3M cell lines expressing MTA1 compared to Res and Pter. As seen in Figure 4A,B, Gnetin C-treated cells demonstrated impaired directional migration compared to Pter (*p* < 0.05) and Res (*p* < 0.01) in both cell lines. The treatment of shMTA1 cells with Res and Pter showed no further reduction in wound-healing capacity, suggesting MTA1-mediated effects, whereas in PC3M cells, the effect of Gnetin C was statistically significant compared to Res and Pter (*p* < 0.05). This indicated a partial MTA1-independent mechanism of cell migration inhibition by Gnetin C (Figure 4A,B).

### 3.4. MTA1 and ETS2 Exhibit Direct Positive Correlation in PCa

**Figure 5:**
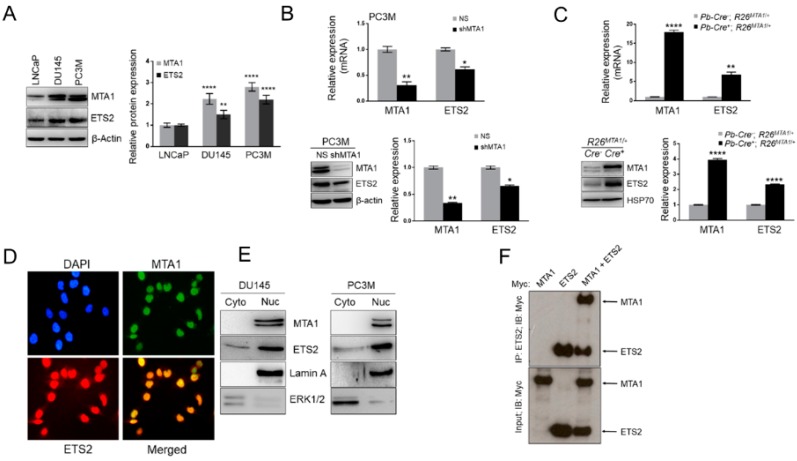
Erythroblastosis E26 transformation-specific 2 (ETS2) expression correlates with MTA1 in PCa. (**A**) ETS2 expression positively correlates with MTA1 expression in human PCa cell lines. (**B**) MTA1 and ETS2 mRNA (*top*) and protein (*bottom*) levels were analyzed in PC3M (NS) and silenced for MTA1 (shMTA1) cells by qRT-PCR and Western blot, respectively. Quantifications represent the mean ±SEM of three independent experiments. * *p* < 0.05; ** *p* < 0.01; **** *p* < 0.0001 (two-way ANOVA). (**C**) Quantitation of MTA1 and ETS2 mRNA (*top*) and protein (*bottom*) levels in prostate tissues (n = 3) of 13-week-old *Pb-Cre*^+^, *R26^MTA1^*^/+^ mice, and Cre-negative normal prostate control. Data represent the mean ±SEM of three prostate tissues. * *p* < 0.05; ** *p* < 0.01; **** *p* < 0.0001 (two-way ANOVA). (**D**) MTA1 and ETS2 protein expression was analyzed by immunofluorescence. Images were pseudo-colored in green to represent MTA1 and in red to represent ETS2. Images show the co-localization of MTA1 and ETS2 (merge, yellow) (magnification x 200). (**E**) Subcellular localization and predominantly nuclear expression of MTA1 and ETS2. Lamin A and Erk1/2 levels were used as a loading control for nuclear and cytoplasmic fractions, respectively. (**F**) MTA1 interacts with ETS2 in an ectopic system. A549 cells were co-transfected with Myc-MTA1, Myc-ETS2, and Myc-MTA1 + Myc-ETS2, and cell lysates were immunoprecipitated with antibodies to ETS2, followed by Western blotting with Myc antibodies.

To determine the mechanism responsible for the MTA1-dependent anticancer efficacy of stilbenes, we interrogated our previously reported MTA1 ChIP-Seq data [11,12,32,36]. Since ETS2 was among the MTA1 target promoters in the MTA1 ChIP-Seq analysis, we hypothesized a possible interrelationship between MTA1 and ETS2. Indeed, we demonstrated a direct association of MTA1 with ETS2 mRNA and protein expression in prostate-specific *Pten* heterozygous mouse prostate tissue as well as in DU145 and LNCaP PCa cells silenced for MTA1 [12]. To further validate the relationship between MTA1 and ETS2, here we examined different PCa cell lines for MTA1 and ETS2 expression, and found that higher MTA1 is associated with higher ETS2 expression in aggressive DU145 and PC3M cells compared to LNCaP (Figure 5A). We further confirmed the link between MTA1 and ETS2 in the most aggressive PC3M cells silenced for MTA1 in addition to previously shown effects in LNCaP and DU145 cells [12]. As shown in Figure 5B, MTA1 knockdown PC3M cells (shMTA1) exhibit the downregulation of ETS2 on both mRNA (Figure 5B, *top*) and protein (Figure 5B, *bottom*) levels. Moreover, the prostate tissues of prostate-specific MTA1 transgenic mice (Pb-Cre^+^; R26^MTA1/+^) [11,36] showed significantly higher ETS2 expression at both mRNA and protein levels compared to a normal prostate (Pb-Cre^−^; R26^MTA1/+^) (Figure 5C). We next performed immunofluorescence experiments using DU145 cells, which revealed that MTA1 and ETS2 co-reside in the nuclei of the cells (Figure 5D). In addition, subcellular fractionation showed predominant nuclear localization for both MTA1 and ETS2 in DU145 and PC3M cells, suggesting a possible interaction between these two proteins (Figure 5E). Therefore, we next performed immunoprecipitation (IP) experiments with an ectopic overexpression of MTA1 and ETS2 by the co-transfection of myc-MTA1 and myc-ETS2 in A549 cells, which revealed interaction between MTA1 and ETS2 (Figure 5F). However, reciprocal co-immunoprecipitation (Co-IP) analysis for interaction between endogenous MTA1 and ETS2 in PC3M cells, which have very high levels of expression for both proteins, revealed no detectable interaction between MTA1 and ETS2 (data not shown).

To explore the clinical significance of the MTA1 and ETS2 interrelationship in PCa, we analyzed the expression of MTA1 and ETS2 in a publicly available PCa patient dataset [38] from the Oncomine database. We found a significantly higher level of ETS2 in tumor samples compared to normal prostate tissues (Supplementary Figure S2A). Although only eight PCa patient samples had information on both MTA1 and ETS2 expression, MTA1 and ETS2 exhibited a significant and strong positive correlation (*p* = 0.0007; r = 0.935) (Supplementary Figure S2B).

### 3.5. ETS2 Participates in MTA1-Mediated Functions in PCa

**Figure 6:**
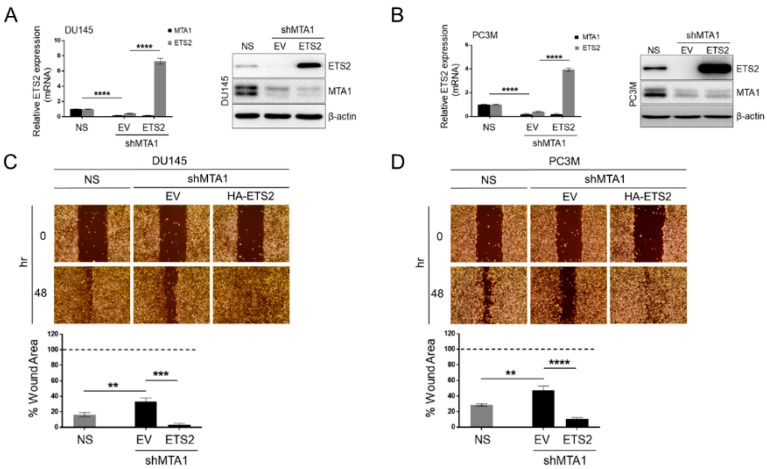
MTA1-mediated mobility of PCa cells is dependent on ETS2. (**A**,**B**) ETS2 overexpression in MTA1 knockdown DU145 and PC3M cells: *left*, mRNA levels were assessed by qRT-PCR; *right,* protein levels were assessed by Western blot. β-actin is a loading control. Data represent the mean ± SEM of three independent experiments. * *p* < 0.05; **p < 0.01; *** *p* < 0.001; **** *p* < 0.0001 (two-way ANOVA). (**C**,**D**) Representative images showing migration ability of DU145 and PC3M MTA1 expressing (NS) and MTA1 knockdown (shMTA1) cells overexpressing ETS2. *Bottom,* quantitation of wound widths as percentage of wound area using ImageJ software is shown for each cell line. Data represent the mean ± SEM of six separate wounds and three independent experiments. * *p* < 0.05; ** *p* < 0.01; *** *p* < 0.001; **** *p* < 0.0001 (one-way ANOVA).

To investigate the role of ETS2 in the MTA1-mediated effects in PCa, we ectopically overexpressed ETS2 in DU145 and PC3M MTA1 knockdown cells by transient transfection of the HA-ETS2 plasmid. Quantitative RT-PCR and Western blots showed the successful overexpression of ETS2 in these cells (Figure 6A,B). As expected, the knockdown of MTA1 led to a significant reduction of cell migration. The overexpression of ETS2 reversed reduced cell migration in shMTA1 cells, indicating that MTA1 regulates cell migration through ETS2 (Figure 6C,D).

### 3.6. ETS2 Inhibition by Gnetin C in PCa Cells is More Potent than Resveratrol and Pterostilbene and is MTA1 Dependent

**Figure 7:**
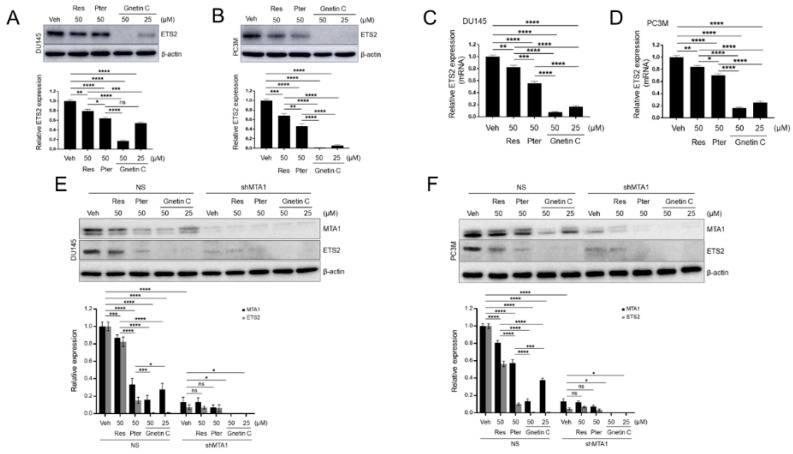
Gnetin C is more potent than resveratrol and pterostilbene in inhibiting MTA1-dependent ETS2 in PCa cells. (**A**,**B**) Comparison of ETS2 inhibition by Res (50 μM), Pter (50 μM), and Gnetin C (25 μM, 50 μM) in DU145 and PC3M cells, respectively. *Bottom*, quantitation of ETS2 levels under Res, Pter, and Gnetin C treatment. (**C**,**D**) ETS2 mRNA levels were analyzed by qRT-PCR after treatment with Res (50 μM), Pter (50 μM), and Gnetin C (25, 50 μM) in DU145 and PC3M cells, respectively. Data represent the mean ± SEM of three independent experiments. * *p* < 0.05; ** *p* < 0.01; *** *p* < 0.001; **** *p* < 0.0001 (one-way ANOVA). (**E**,**F**) MTA1 and ETS2 protein levels were determined in DU145 and PC3M cells expressing MTA1 (NS) and MTA1 knockdowns (shMTA1) treated with Res (50 μM), Pter (50 μM), and Gnetin C (25,50 μM). *Bottom*, quantifications of MTA1 and ETS2 levels under Res, Pter, and Gnetin C treatment in DU145 and PC3M cells. Data represent the mean ± SEM of three independent experiments. * *p* < 0.05; ** *p* < 0.01; *** *p* < 0.001; **** *p* < 0.0001 (two-way ANOVA).

To extend our observation on stilbene-mediated anticancer pathways, we measured the effects of Gnetin C on ETS2 expression. We found that when compared to Res and Pter applied at the same 50-μM concentration, Gnetin C demonstrated more potent inhibition of the ETS2 protein and mRNA levels in both DU145 and PC3M cells (Figure 7A–D). Notably, Gnetin C demonstrated significant inhibition of ETS2 at a 25-μM dose, which was twofold lower (Figure 7A–D).

To examine the MTA1-mediated downregulation of ETS2 by stilbenes, we treated DU145 and PC3M control non-silenced (NS) and MTA1 knockdown (shMTA1) cells with Res, Pter, and Gnetin C. As expected, MTA1 silencing led to a significant downregulation of ETS2 (Figure 7E,F). As seen in Figure 7E,F, most of the ETS2 downregulation in cells expressing MTA1 (NS) correlated with MTA1 downregulation by stilbenes, and was more pronounced with Gnetin C treatment. However, there was a further slight inhibition of ETS2 by stilbenes in MTA1 knockdown cells, suggesting a degree of MTA1 independence for ETS2 inhibition in PCa cells.

## 4. Discussion

Pharmacologically safe dietary compounds with targeted anticancer activity are of particular interest for chemoprevention and therapy in cancer. We have previously shown that resveratrol and pterostilbene specifically inhibit MTA1 and MTA1-guided tumor promoting signaling in PCa [9,12,28,29,30,32].

MTA1 belongs to the MTA family of proteins that are part of the nucleosome remodeling and deacetylase (NuRD) multiprotein complex, which is involved in chromatin remodeling and transcriptional regulation [10,39]. We and others have reported on the critical role of MTA1 in PCa pathogenesis, progression, and metastasis [7,8,9,10,11,12,28,29,30,32,36], signifying MTA1 signaling as a valuable therapeutic target in PCa. Moreover, we have identified pharmacological inhibitors of MTA1 including dietary stilbenes such as resveratrol and pterostilbene, and reported their potent MTA1-mediated anticancer effects in PCa [9,12,28,29,30,32,37]. Therefore, it is evident that further development of MTA1-targeted chemoprevention and therapeutic strategies in PCa are needed.

Gnetin C is a resveratrol dimer that has been detected in the fruits, leaves, and seeds of melinjo, which is an edible plant native to Southeast Asia and the western Pacific Islands [40]. In vitro and in vivo anticancer properties of pure Gnetin C as well as melinjo seed extract (MSE) have been reported, and nontoxic effects of both Gnetin C and MSE in nonmalignant cells (HEK-293T kidney and RWPE1 prostate cells) were demonstrated [41,42,43,44]. Moreover, the ability of extract to target specific pathways, namely ERK1/2 and AKT/mTOR, was also identified in a mouse model of tumor angiogenesis [41] and in acute myeloid leukemia (AML) xenografts [43]. Importantly, MSE appears to be safe in high doses (1000 mg/kg/day) in rats [45], mice (50–100 mg/kg) [42], and humans (750–5000 mg daily) [46,47]. Moreover, no adverse events were reported during 2 weeks of daily pure Gnetin C consumption (150 mg/day) in healthy volunteers [48]. Vitally, Gnetin C has improved pharmacokinetics, including higher bioavailability, compared to resveratrol [47,49].

Our observation of MTA1 inhibition by resveratrol and pterostilbene linked with known improved pharmacokinetics of Gnetin C posed three major questions. First, does Gnetin C inhibit MTA1 more potently than resveratrol and pterostilbene? Second, does Gnetin C exhibit superior MTA1-mediated anticancer efficacy than resveratrol and pterostilbene? Third, what is the MTA1-associated mechanism that is responsible for the enhanced anticancer activity of Gnetin C?

In this study, we have presented evidence that Gnetin C inhibits MTA1 more potently than resveratrol and even pterostilbene, which was considered the most potent MTA1 inhibitory stilbene until now. In our functional experiments, we have demonstrated the supreme efficacy of MTA1-mediated cytotoxic, pro-apoptotic, anti-invasion, and anti-migratory effects of Gnetin C in two aggressive PCa cell lines compared to resveratrol and pterostilbene. In addition, we identified a more potent MTA1-associated downregulation of ETS2 by Gnetin C compared to resveratrol and pterostilbene to be responsible for its superior MTA1-mediated antitumor efficacy. Of note, although the anticancer activity of Gnetin C in prostate cancer cells was mostly MTA1-dependent, we also observed MTA1-independent effects in our molecular and functional studies.

While we have reported a positive relationship between MTA1 and ETS2 in this study and previously [12], suggesting a tumor-promoting role for ETS2, the literature evidence remains divided, suggesting both tumor-promoting and tumor-suppressive functions for ETS2 [13,14,15,16,17,18,19,20,21,22,23,24,25,26,27]. The oncogenic/tumor-promoting role of ETS2 is supported by studies showing that a blockade of ETS2 not only reduces the transformed properties of DU145 and PC3 cells [13], but also induces apoptosis (reduced levels of the anti-apoptotic protein bcl-xL) and inhibits growth (reduced levels of cyclin D1 and c-Myc) [14], revealing a specific role of ETS2 in promoting the growth and survival of PCa cells. ETS2 has also been shown to be a prostate basal cell marker that is overexpressed in some carcinomas [24]. The association between ETS2 and mutant p53 resulting in the recruitment of mutant p53 to promoters of genes that stimulate drug resistance, migration, invasion, and metastasis has also been documented [25,26,27]. On the other hand, some studies have demonstrated that (i) the ectopic expression of ETS2 decreased the proliferation and invasion of PCa cell lines [15,16] and (ii) a reduced expression of ETS2 was associated with biochemical recurrence and lethal disease [17], suggesting a tumor-suppressive role for ETS2. The role of ETS2 as a tumor suppressor is also supported by studies showing that PCa with TMPRSS2-ERG fusions generated through the deletion of a region containing ETS2 among other genes, represent a more aggressive and hormone-refractory disease [18,20]. However, the debate is kept alive by reports demonstrating both significantly low [21] and significantly high [19,22] expression of ETS2 in human PCa tissue samples. Currently, it is largely accepted that ETS2 can act as an oncogene in some cellular backgrounds and as a tumor suppressor in others. Moreover, specific molecular mechanisms may allow ETS2 to switch between oncogenic and tumor suppressive functions in a cell-type and genetic context-specific manner [23].

In summary, our results provide a plausible mechanism for the superior MTA1-mediated anticancer effects of Gnetin C in PCa. To the best of our knowledge, this is a first report demonstrating that Gnetin C acts an effective anticancer agent in PCa, at least in part, through inducing the MTA1-dependent and independent downregulation of ETS2.

## 5. Conclusions

We report herein that Gnetin C, a resveratrol dimer and dietary compound found in the melinjo plant, is more potent than resveratrol and pterostilbene in exerting its anticancer activity in PCa, at least in part, through the inhibition of cancer-promoting co-operation between MTA1 and ETS2. Our data indicate that Gnetin C causes cytotoxicity, cell death, and a reduction of the metastatic ability of PCa cells through MTA1-mediated mechanisms. Although the effects of Gnetin C are mainly mediated through MTA1 and the MTA1-dependent inhibition of ETS2, Gnetin C also demonstrated MTA1-independent targeting of ETS2. Therefore, the dual inhibition of the MTA1/ETS2 axis by Gnetin C may provide more robust anticancer effects in PCa. Taken together, our findings implicate the potential of Gnetin C as a MTA1/ETS2-targeted chemopreventive and possibly therapeutic strategy in PCa.

## Supplementary Materials

The following are available online at https://www.mdpi.com/2072-6643/11/9/2096/s1, Figure S1: Gnetin C is the most potent inducer of cell death assessed by flow cytometry in DU145 and PC3M cells. Sub-G1 population of dead cells is indicated in each panel, Figure S2: Meta-analysis for MTA1 and ETS2 expression in publicly available PCa patient dataset^35^ using Oncomine database. (A) ETS2 expression is higher in human PCa compared to normal and (B) ETS2 correlates with MTA1 expression in human PCa, Table S1: Primary antibodies used in this study, Table S2: Primers for qRT-PCR used in this study.
