# Supplementary material for: MTA1-Dependent Anticancer Activity of Gnetin C in Prostate Cancer
Source: Nutrients. 2019 Sep 4;11(9):2096. doi: 10.3390/nu11092096 (PMC6770780; doi:10.3390/nu11092096)
Supplement: Supplementary file 1 [file nutrients-11-02096-s001.pdf]

Supplementary Information

“MTA1-dependent anticancer activity of Gnetin C in prostate cancer”

Avinash Kumar, Kshiti Dholakia, Gabriela Sikorska, Luis A Martinez, and Anait S. Levenson

Table S1. Primary antibodies used in this study.

Antibody	Method	Dilution	Source	Catalog#
MTA1	Immunoblot	1:1000	Cell Signaling Technologies	5647
	Immunofluorescence	1:50	Santa Cruz Biotechnology	sc-17773
ETS2	Immunoblot	1:200	Santa Cruz Biotechnology	sc-365666
	Immunofluorescence	1:50	Santa Cruz Biotechnology	sc-365666
Myc	Immunoblot	1:500	Santa Cruz Biotechnology	sc-40
β-actin	Immunoblot	1:2500	Santa Cruz Biotechnology	sc-69879

Table S2. Primers for qRT-PCR used in this study.

Primers	Sequence
MTA1 forward	5'- AGC TAC GAG CAG CAC AAC GGG GT - 3'
MTA1 reverse	5'- CAC GCT TGG TTT CCG AGG AT - 3'
ETS2 forward	5'- GGG AGT TCA AGC TTG CTG AC - 3'
ETS2 reverse	5'- CCC GAA GTC TTG TGG ATG AT - 3'
β-actin forward	5'- CGT GGG CCG CCC TAG GCA CCA - 3'
β-actin reverse	5'- TTG GCT TAG GGT TCA GGG GGG - 3'

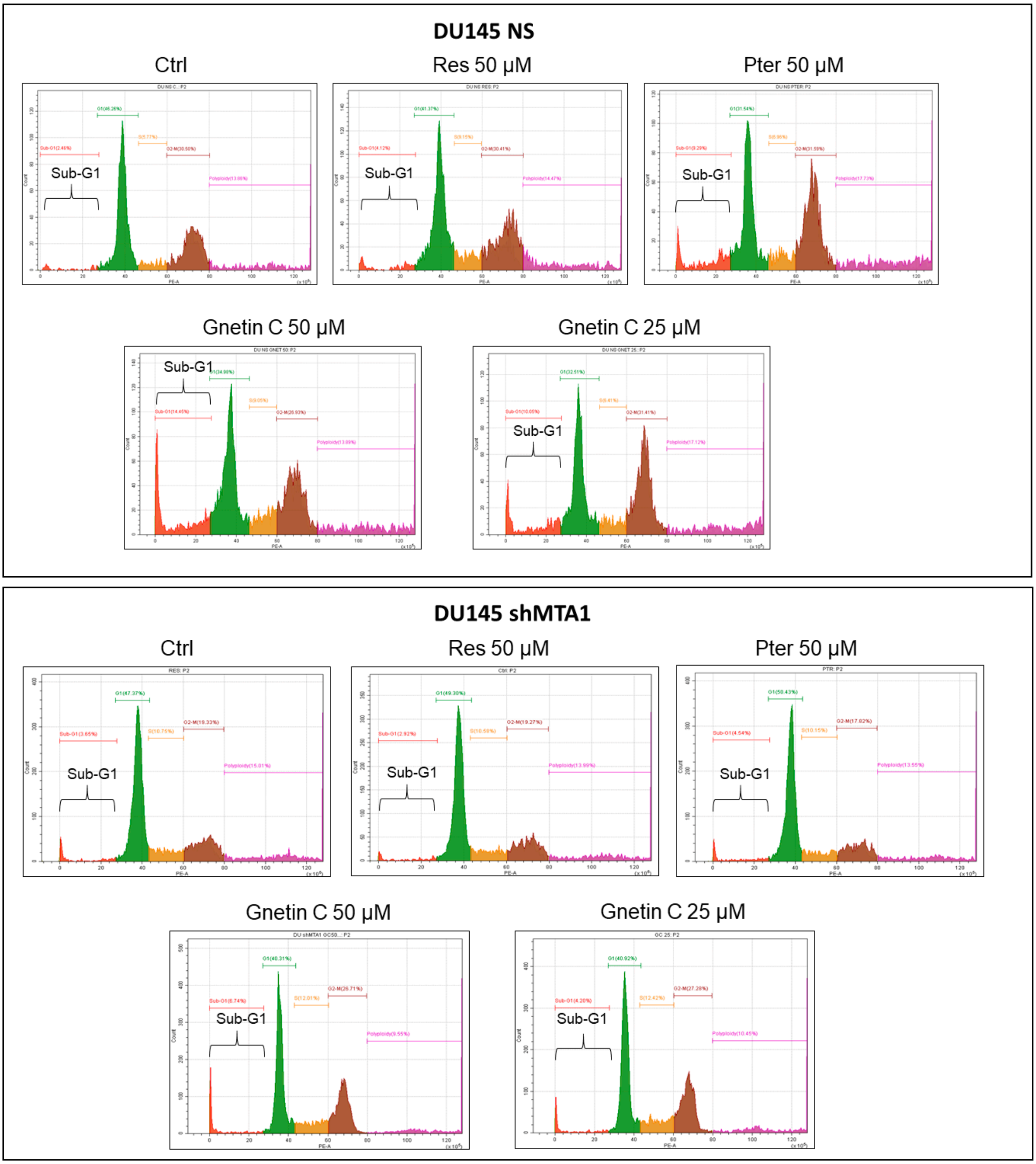
DU145 NS
Ctrl
Res 50 μM
Pter 50 μM
Sub-G1
Sub-G1
Sub-G1
Gnetin C 50 μM
Gnetin C 25 μM
Sub-G1
Sub-G1
DU145 shMTA1
Ctrl
Res 50 μM
Pter 50 μM
Sub-G1
Sub-G1
Sub-G1
Gnetin C 50 μM
Gnetin C 25 μM
Sub-G1
Sub-G1

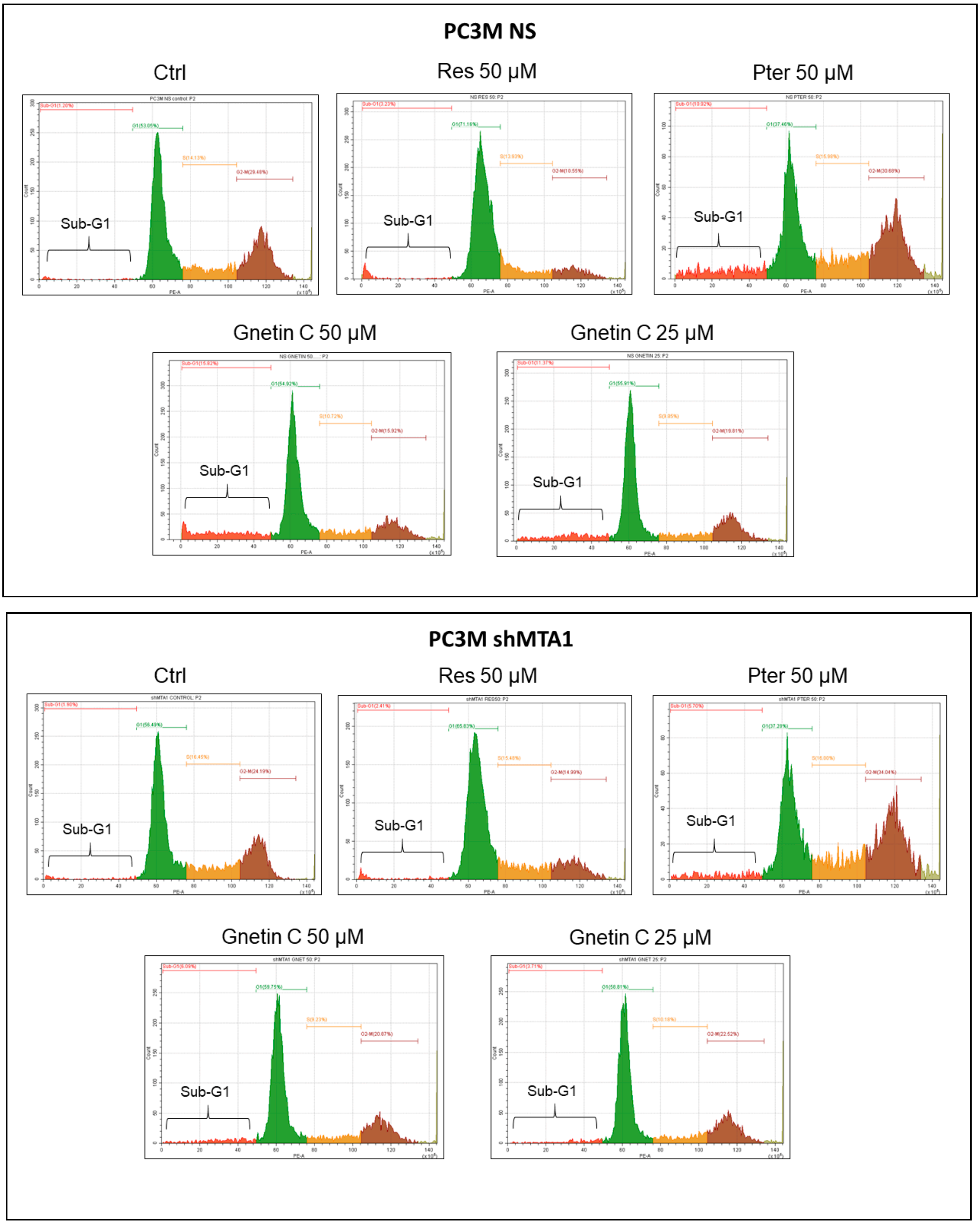

Figure S1. Gnetin C is the most potent inducer of cell death assessed by flow cytometry in DU145 and PC3M cells. Sub-G1 population of dead cells is indicated in each panel.

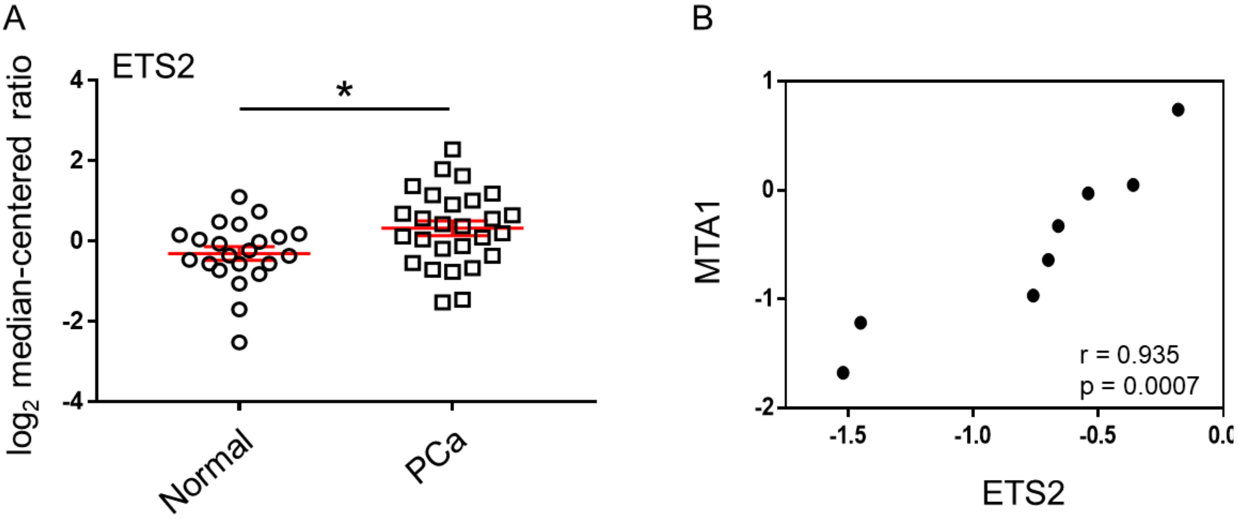

Figure S2. Meta-analysis for MTA1 and ETS2 expression in publicly available PCa patient dataset[35] using Oncomine database. A) ETS2 expression is higher in human PCa compared to normal and B) ETS2 correlates with MTA1 expression in human PCa.
